# I_H_ activity is increased in populations of slow versus fast motor axons of the rat

**DOI:** 10.3389/fnhum.2014.00766

**Published:** 2014-09-25

**Authors:** Chad Lorenz, Kelvin E. Jones

**Affiliations:** ^1^Faculty of Physical Education and Recreation, University of AlbertaEdmonton, AB, Canada; ^2^Neuroscience and Mental Health Institute, University of AlbertaEdmonton, AB, Canada

**Keywords:** axon physiology, ion channels, hyperpolarization-activated inwardly rectifying cation conductance, nerve excitability test

## Abstract

Much is known about the electrophysiological variation in motoneuron somata across different motor units. However, comparatively less is known about electrophysiological variation in motor axons and how this could impact function or electrodiagnosis in healthy or diseased states. We performed nerve excitability testing on two groups of motor axons in Sprague–Dawley rats that are known to differ significantly in their chronic daily activity patterns and in the relative proportion of motor unit types: one group innervating the soleus (“slow motor axons”) and the other group innervating the tibialis anterior (“fast motor axons”) muscles. We found that slow motor axons have significantly larger accommodation compared to fast motor axons upon application of a 100 ms hyperpolarizing conditioning stimulus that is 40% of axon threshold (*Z* = 3.24, *p* = 0.001) or 20% of axon threshold (*Z* = 2.67, *p* = 0.008). Slow motor axons had larger accommodation to hyperpolarizing currents in the current-threshold measurement (-80% *Z* = 3.07, *p* = 0.002; -90% *Z* = 2.98, *p* = 0.003). In addition, we found that slow motor axons have a significantly smaller rheobase than fast motor axons (*Z* = -1.99, *p* = 0.047) accompanied by a lower threshold in stimulus-response curves. The results provide evidence that slow motor axons have greater activity of the hyperpolarization-activated inwardly rectifying cation conductance (I_H_) than fast motor axons. It is possible that this difference between fast and slow axons is caused by an adaptation to their chronic differences in daily activity patterns, and that this adaptation might have a functional effect on the motor unit. Moreover, these findings indicate that slow and fast motor axons may react differently to pathological conditions.

## INTRODUCTION

Many studies have demonstrated that changes in chronic daily activity patterns alter the electrophysiological properties of cat and rodent motor neurons measured from the soma: after exercise (Beaumont and Gardiner, 2002, 2003; Gardiner et al., 2005), chronic stimulation (Munson et al., 1997), and hindlimb unweighting or spinal transections (Hochman and McCrea, 1994; Cormery et al., 2005). Muscle contractile properties also change in response to different activity levels like chronic stimulation and exercise (Kernell et al., 1987; Westgaard and Lomo, 1988; Gordon et al., 1997) or decreased activity levels through spinal transection (Cope et al., 1986; Munson et al., 1986). However, there is comparatively little known about the association between the electrophysiological properties of motor axons and motor unit type, or whether the axons change in response to altered chronic activity levels that induce plasticity of motor unit phenotype. The primary exception is conduction velocity (CV), which is altered in response to chronic changes in daily activity patterns of the motor units (Carp and Wolpaw, 1994; Munson et al., 1997; Beaumont and Gardiner, 2002). However, it has been acknowledgd for some time that conventional nerve CV provides limited physiological insight and additional measures from nerve excitability studies are warranted (Krarup and Moldovan, 2009; Kiernan and Lin, 2012).

Nerve excitability studies in humans have demonstrated that electrophysiology is different between axons innervating different muscles (Kuwabara et al., 2000, 2001; Krishnan et al., 2004; Bae et al., 2009; Jankelowitz and Burke, 2009) and between motor axons innervating the same muscle but activated at different percutaneous stimulation thresholds (Trevillion et al., 2010). Similar studies in rodents suggested that motor axons activated at different stimulation thresholds vary moderately in measures of nerve excitability (Mori et al., 2010; Nodera and Rutkove, 2012). These studies suggest that motor axon conductances such as hyperpolarization-activated inwardly rectifying cation conductance (I_H_) and slow potassium conductance (I_Ks_) may vary across muscles and axons of different threshold. However, none of these studies have examined populations of axons from muscles with distinct distributions of motor unit types. The previous research suggests that the major differences in motor axons are a result of differences in conductances rather than simply a consequence of different axon diameters.

The purpose of this study is to compare the electrophysiology of axons innervating the soleus muscle (SOL) to those innervating the tibialis anterior (TA) muscle in the rat. SOL axons were considered to represent a population of *slow-* and TA axons *fast-* motor axons. About 94% of rat TA motor units are classified as a fast (fast fatigable or fast fatigue-resistant) motor unit type while 6% are slow, based on a modified version of Burke’s criteria originally used to distinguish motor units in the cat hindlimb (Totosy de Zepetnek et al., 1992). Note the motor unit distribution in the human TA has a much larger contribution from slow motor units (Johnson et al., 1973). On the other hand, about 80% of the motor units in rat SOL are classified as slow motor unit type while 20% are the fast motor unit type (Gillespie et al., 1987). Rat TA and SOL motor units have dramatically different activity patterns in locomotion (Gorassini et al., 2000) and presumably much different overall daily activity patterns (e.g., Hennig and Lomo, 1985). Thus, by using TA and SOL axons in this study, we analyzed axons of different motor unit types as well as axons with different chronic daily firing patterns.

## MATERIALS AND METHODS

### SURGERY AND RECORDINGS

A total of 14 female Sprague–Dawley rats, weighing 280 ± 50 g (mean ± SD), were used in this study. These weights in female Sprague–Dawley rats correspond to an age of approximately 90 days, which represents young but sexually mature females (Yang et al., 2000; George and Bostock, 2007). Rats were housed in pairs in a 12:12 h light–dark cycle, environmentally controlled (22–24°C, 40–70% humidity) room. Water and rat chow (Lab Diet 5001, PMI Nutrition, Brentwood, MO, USA) were provided *ad libitum*. All animal studies were conducted in accordance with the Canadian Council on Animal Care Guidelines and Policies with approval from the Animal Care and Use Committee: Health Sciences for the University of Alberta.

Anesthesia was induced by intraperitoneal injection of a mixed dose of 60 mg/kg ketamine and 7.2 mg/kg xylazine (KX) with additional KX dosages to maintain a surgical plane of anesthesia for the duration of the experiment. A rectal thermometer monitored internal body temperature, which was maintained between 34.5 and 38.5°C by a heating lamp. Upon completion of all experiments, animals were euthanized by an overdose of KX followed by cervical dislocation.

**FIGURE 1:**
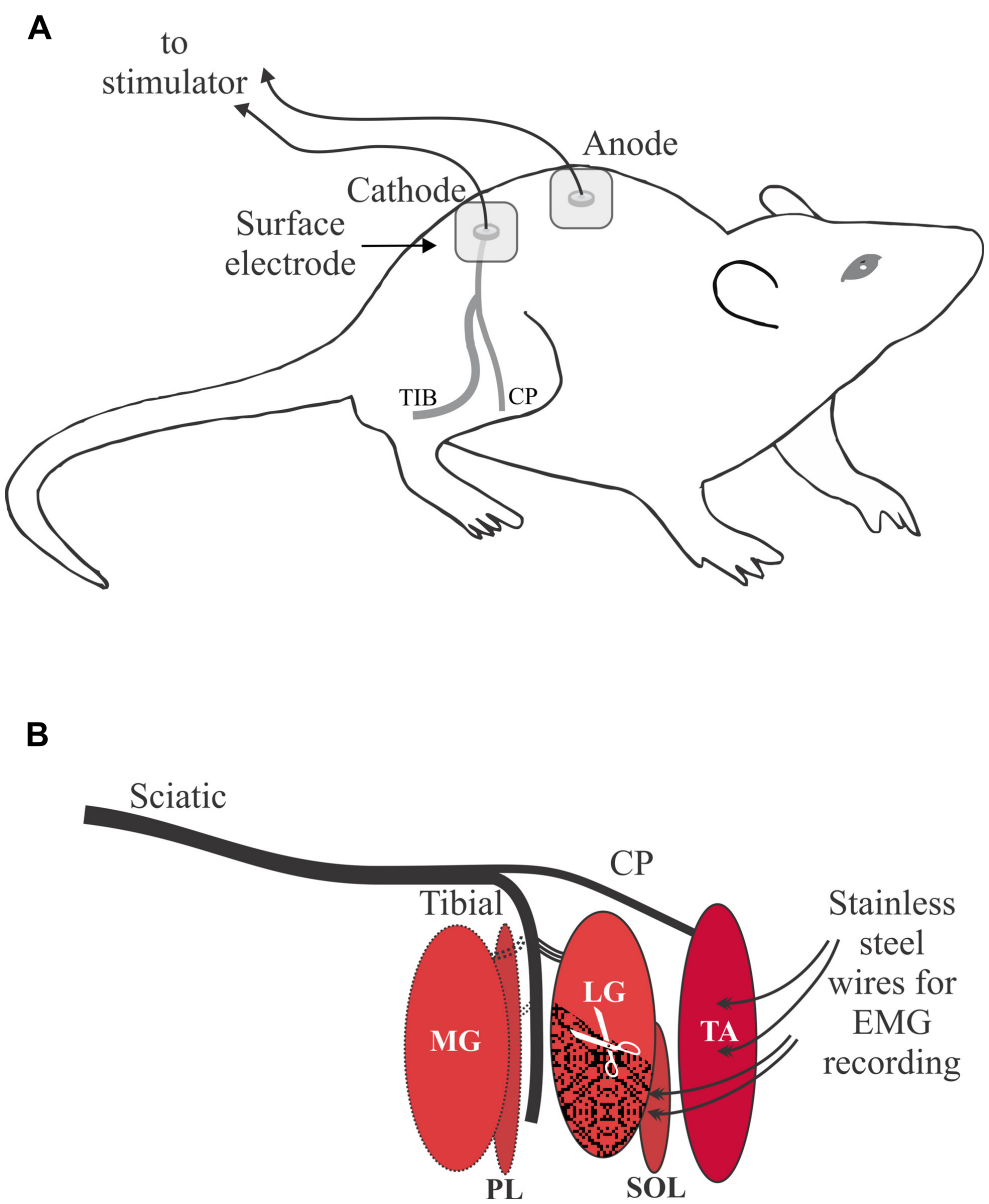
**Overview of the experimental setup. (A)** Percutaneous electrical stimulation of the sciatic nerve with the cathode over the sciatic notch illustrated for the right leg. Proper skin preparation was essential under all stimulating electrodes. **(B)** Schematic of the branching of the sciatic nerve to the two target muscles: tibialis anterior (TA) and soleus (SOL). Other muscles of the lower leg were denervated: medial gastrocnemius (MG), plantaris (PL). The distal portion of the lateral gastrocnemius (LG) muscle was excised to reduced artifact from LG into electromyographic (EMG) recordings from SOL.

An electrical clipper was used to initially remove hair over the lower back and legs followed by a depilatory cream to remove the remaining hair on the lower back and hip areas. Percutaneous electrical stimulation was delivered via Ag/AgCl 3M Red Dot electrodes (10 mm diameter), with the active electrode placed over the sciatic notch and the return electrode placed over the lumbar vertebrae at the midline of the back (**Figure 1A**). Electromyographic (EMG) recordings were made using Teflon-coated stainless steel monofilament wire (Cooner Wire 765–40; 40G) with approximately 3 mm of Teflon removed from the end of the wire. The wire was threaded through a hypodermic needle (TA 26G, SOL 27G) with ∼2 mm extending from the tip and twisted to form a hook. To record a bipolar intramuscular compound muscle action potential (CMAP) signal in TA the first needle was inserted near the motor endplate on the proximal 1/3 of the muscle, and a second needle was inserted about 2.5 mm distally. Insertion of the hypodermic needles in SOL was similar except they were placed about 5 mm apart because of the orientation of the motor endplate and muscle fibers in SOL. After the stainless steel recording electrodes were securely placed in each muscle, the needles were removed. To minimize EMG from other muscles and contamination of the target CMAP during the threshold tracking procedure, extensor digitorum longus, flexor digitorum longus, medial gastrocnemius, and plantaris were denervated. The distal 3/5 of lateral gastrocnemius (LG) was excised, rather than denervated, because SOL axons are intertwined with LG axons in a shared nerve branch that is not easily separated in the rat (**Figure 1B**).

Compound muscle action potentials were amplified (to cover one-half to two-thirds of the A/D range), filtered (10–1000 Hz) and digitized by a 12-bit A/D board (National Instruments DAQ-6062e, Austin, TX, USA) at a rate of 10 kHz. Noise introduced from nearby power sources was removed in real time using a Hum Bug 50/60 Hz Noise Eliminator (Quest Scientific Instruments, North Vancouver, BC, Canada). In cases of excessive levels of noise which occurred in six rats, a 60 Hz notch filter was also implemented. The time required from anesthetic induction to complete all measures in an animal was typically 1 h. In five animals the electrodes were repositioned and the tests repeated as a result of signal contamination. The time required in these cases was approximately 1.5 h. Arterial blood gases and acid base status were not monitored but are well maintained at rest in this preparation over this time period (Jendzjowsky and DeLorey, 2013).

### NERVE EXCITABILITY TESTING

Multiple excitability measures of TA and SOL axons were made from the right leg. Stimuli were delivered using QTRAC software (Institute of Neurology, Queen Square, London, UK) and an isolated bipolar constant current stimulator (Digitimer DS5, Digitimer Ltd., Welwyn Garden City, Hertfordshire, UK). The QTRAC protocol “TRONDCMW” consists of five nerve excitability indices: threshold electrotonus (TE), current-threshold (I/V), recovery cycle (RC), rheobase, and strength-duration time constant (SDTC). The primary outcome measure for each of these indices is the change in the amplitude of the test stimulus required to produce a target CMAP: in this case 40% of the maximum with a tolerance of ±7.5%. The duration of the test stimulus was 1.0 ms except during rheobase and SDTC in which the duration varies between 0.2 and 1.0 ms. Krishnan et al. (2009) has a more detailed description of this validated methodology.

For TE, a 100 ms sub-threshold conditioning stimulus with an amplitude expressed as a percentage (±40 and ±20%) of the test stimulus is applied and the change in test stimulus amplitude is measured at multiple delays relative to the start of the conditioning stimulus. The I/V is a similar test with a 200 ms sub-threshold conditioning stimulus and the change in test stimulus amplitude is measured at the end of the conditioning stimulus. The amplitude of the conditioning stimulus for I/V measurements varies from +50 to -100% of the unconditioned test stimulus. In RC, a supramaximal conditioning stimulus is applied to the nerve, and the test stimulus is applied at delays ranging from 2 to 200 ms. Rheobase and SDTC are determined via linear regression of the charge-duration data obtained through five test stimuli that range in duration from 0.2 to 1.0 ms.

### ANALYSIS

All statistical analysis was done in SPSS version 21. Normality was assessed by the Shapiro–Wilk test and it was found that most data groups (groups within each of the five nerve excitability indices) *violated* the normality assumption at an alpha level of 0.05. Specifically, the hypothesis of a normal distribution was rejected for all data groups except TE +20% and depolarizing I/V measures. Therefore the non-parametric Wilcoxon signed-rank test was used for paired comparison of TA and SOL axons within each individual rat. For comparisons involving TE, paired comparison of TA and SOL axons were made at three delays for each of the four conditioning stimulus levels, i.e., ±40 and ±20%. For comparisons involving I/V, there were five data groups analyzed from I/V +50 to +10% and 10 groups analyzed from I/V 10 to -100%, all spaced by increments of 10%. In RC, all 18 delays ranging between 2.0 and 200 ms delay were analyzed. Finally, rheobase and SDTC were also used to compare TA and SOL axons. In order to control for type I statistical errors, we divided the alpha level (0.05) by the number of data groups within each respective nerve excitability measure. Therefore, the adjusted alpha level (α_a_) for determining statistical significance was 0.017 in depolarizing and hyperpolarizing TE, 0.01 in depolarizing I/V, 0.005 in hyperpolarizing I/V, 0.006 in hyperpolarizing I/V Slope, 0.003 in RC, and 0.05 in rheobase and SDTC. The whiskers in each box-and-whisker plot represent 5 and 95% confidence intervals, while the box represents the interquartile range and the line the median. Line plots include the median value and error bars are the 95% confidence interval.

## RESULTS

### THRESHOLD ELECTROTONUS

**FIGURE 2:**
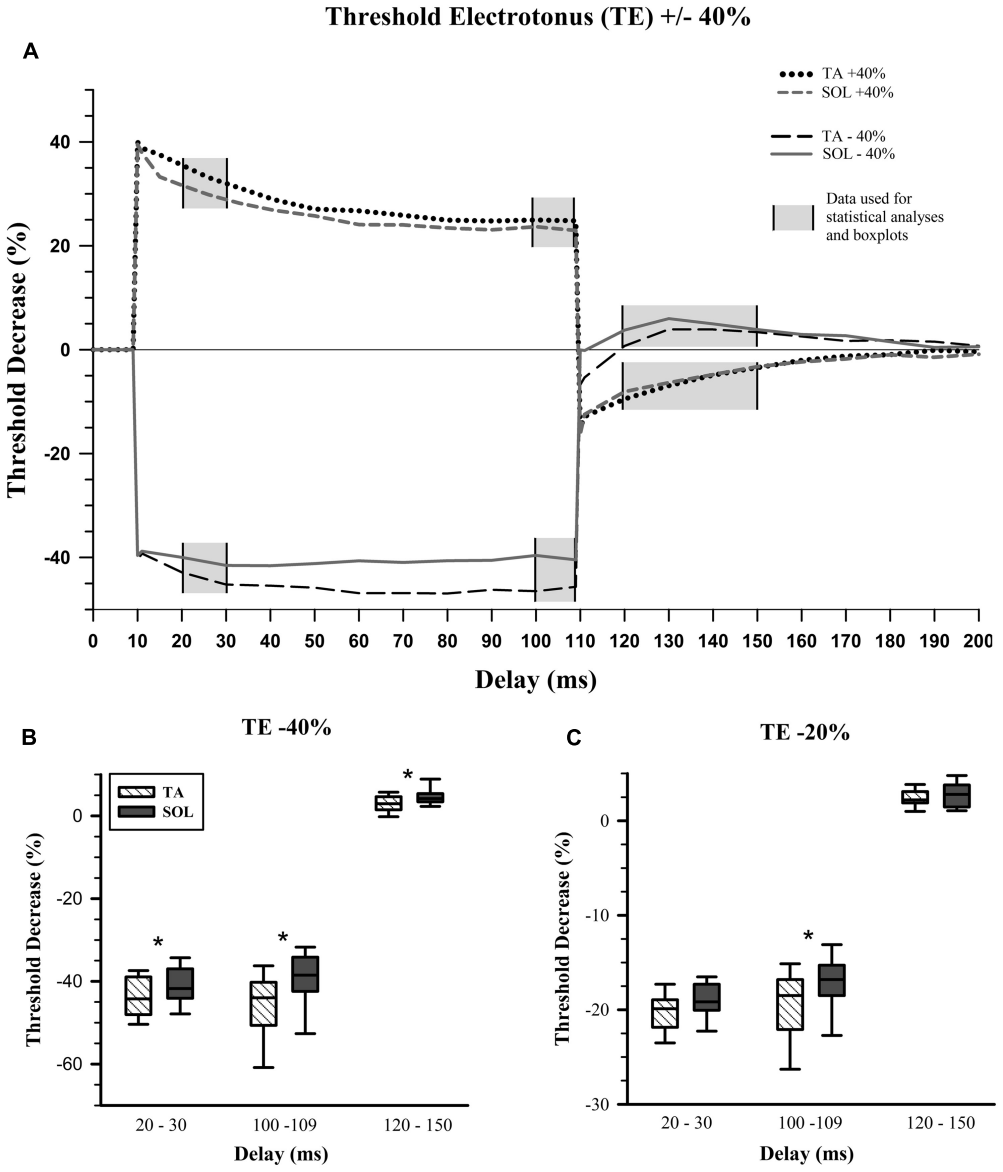
**Threshold electrotonus. (A)** Average values for depolarizing and hyperpolarizing conditioning pulses with 40% amplitude. Planned statistical comparisons of TA and SOL axons were done at delay ranges illustrated with a gray background. **(B)** Percent change in threshold for the hyperpolarizing 40% threshold electrotonus (TE) condition. The whiskers in each box-and-whisker plot represent 5 and 95% confidence intervals, while the box represents the interquartile range and the midline the median. TA data in hatched boxes, SOL in gray boxes. Wilcoxon signed-rank tests, which are paired comparisons, show a statistically significant increase in median “Threshold Decrease (%)” for SOL compared to TA axons at all delays. **(C)** Percent change in threshold for the hyperpolarizing 20% TE condition. There is a statistically significant increase in median threshold change for SOL compared to TA axons, only at the end of the hyperpolarizing condition pulse, i.e., 100–109 ms. ^∗^*p* < 0.017.

Previous studies using nerve excitability testing have indicated that particular testing delays within the TE measurement are of greater interest since these are where properties diverge (Schwarz et al., 2006; George and Bostock, 2007; Sittl et al., 2011). These delays are 20–30, 100–109, and 120–150 ms and constitute the planned contrasts for statistical analysis (**Figure 2A**, gray areas). We averaged all the data within each of these delay ranges and used these values for comparisons between TA and SOL axons. **Figure 2A** displays the averaged data from TA and SOL axons for depolarizing TE +40% and hyperpolarizing TE -40%.

The initial fast phase, which is proportional to the applied current, was the same for TA and SOL axons for all depolarizing and hyperpolarizing conditioning currents. During depolarizing TE +40% there was no statistical difference between TA and SOL axons. In hyperpolarizing TE -40%, TA axons had a significantly greater threshold increase than SOL axons at 20–30 ms (*Z* = 3.24, *p* = 0.001) and 100–109 ms (*Z* = 3.24, *p* = 0.001), and a significantly smaller threshold decrease at 120–150 ms (*Z* = -2.61, *p* = 0.009; **Figure 2B**). For the TE -20% condition at 100–109 ms, TA axons had a significantly greater threshold increase than SOL axons (*Z* = 2.67, *p* = 0.008; **Figure 2C**). The data for these measures are given in **Table 1**.

**Table 1:** Mean values and 95% confidence intervals for the dependent variables generated by nerve excitability testing (NET) of *fast* TA versus *slow* SOL motor axons.

NET measure	TA axons (mean ± 95% CI)	SOL axons (mean ± 95% CI)	*p*-value <α_a_
**Threshold electrotonus (%)**
TE +40% at 20–30 ms	34.35 ± 2.53	30.76 ± 3.09	
TE +40% at 100–109 ms	24.89 ± 2.59	23.32 ± 2.20	
TE +40% at 120–150 ms	-6.22 ± 0.92	-5.63 ± 1.20	
TE -40% at 20–30 ms	-44.04 ± 2.62	-40.75 ± 2.67	*
TE -40% at 100–109 ms	-46.07 ± 4.60	-40.02 ± 3.83	*
TE -40% at 120–150 ms	2.98 ± 1.16	4.65 ± 1.12	*
TE -20% at 20–30 ms	-20.20 ± 1.15	-19.14 ± 1.05	
TE -20% at 100–109 ms	-19.54 ± 2.23	-17.18 ± 1.71	*
TE -20% at 120–150 ms	2.35 ± 0.54	2.66 ± 0.71	
**Current-threshold (%)**
I/ V -80%	-135.15 ± 19.82	-112.89 ± 14.90	*
I/ V -90%	-161.12 ± 21.68	-135.32 ± 17.37	*
I/ V slope at -40%	0.66 ± 0.091	0.79 ± 0.12	*
I/ V slope at -50%	0.56 ± 0.076	0.67 ± 0.095	*
**Recovery cycle (%)**
RC at 2.0 ms	60.07 ± 10.42	96.78 ± 33.34	
RC at 2.5 ms	39.06 ± 6.95	58.46 ± 17.10	
RC at 3.2 ms	22.56 ± 5.22	31.54 ± 14.68	
RC at 4.0 ms	8.66 ± 5.40	19.49 ± 9.25	
RC at 5.0 ms	6.46 ± 3.54	10.78 ± 3.46	
RC at 6.3 ms	5.64 ± 2.82	8.06 ± 2.37	
**Rheobase (mA) and strength-duration time constant (ms)**
Rheobase	1.45 ± 0.20	1.39 ± 0.34	*
SDTC	0.25 ± 0.032	0.28 ± 0.040	

*The alpha criterion was adjusted (α_a_) for multiple comparisons for each of the main outcome measures. TE = 0.017, depolarizing I/V = 0.01, hyperpolarizing I/V = 0.005, I/V slope = 0.006, RC = 0.003, and 0.05 for rheobase and SDTC. ^∗^ indicates *p*-values less than these adjusted alpha.*

### CURRENT-THRESHOLD

**FIGURE 3:**
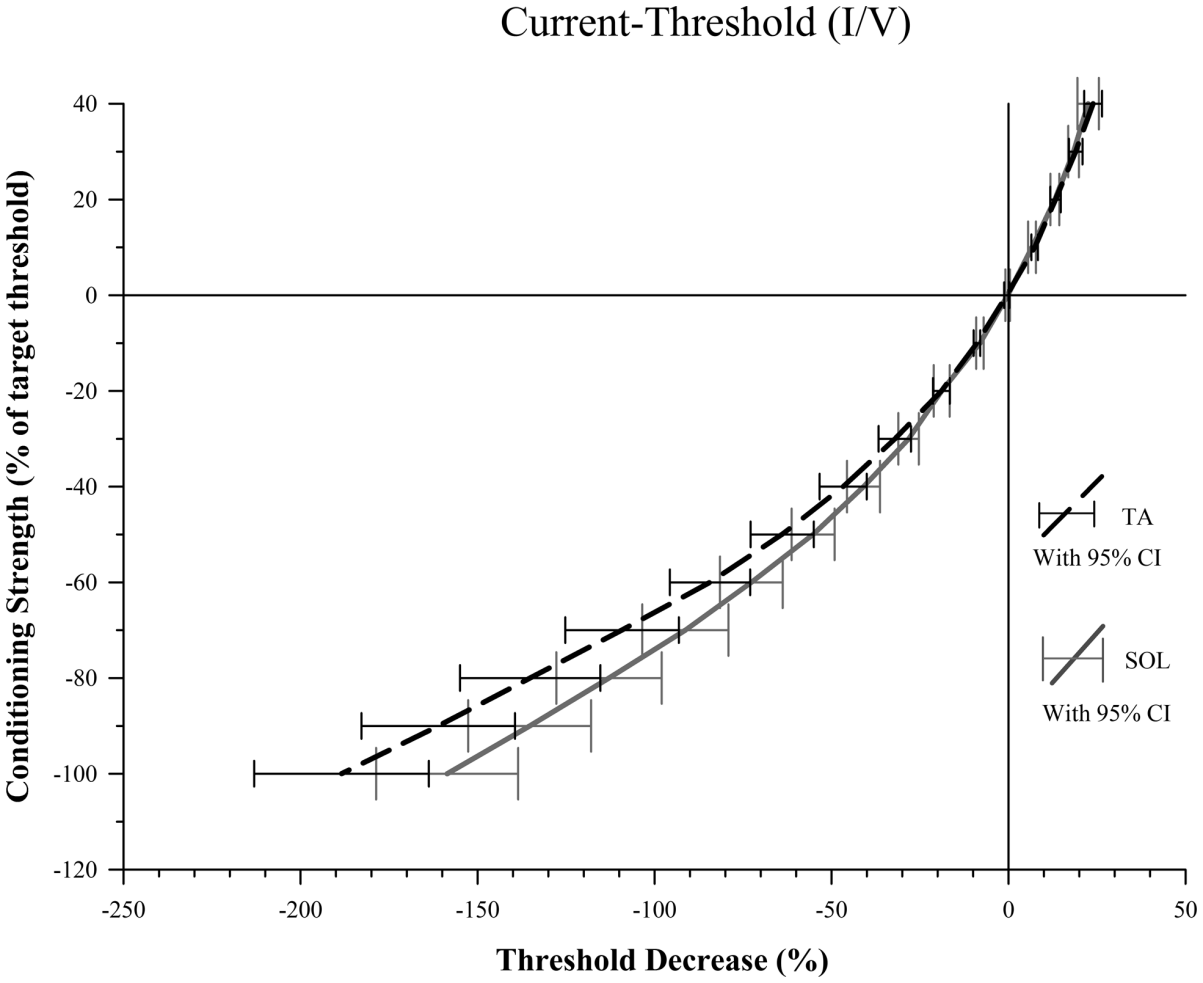
**Conditioning current versus threshold.** For conditioning strengths of -80 and -90% there is a significantly smaller decrease in threshold for SOL compared to TA axons indicative of stronger depolarization current activated by the hyperpolarization for SOL axons. The difference between percent threshold decrease of SOL and TA axons at -80% was 22.3, 25.8 at -90% and 29.8 at a conditioning strength of -100%. Increased variance at -100% likely contributed to the failure to meet the threshold alpha of 0.006 to reject the null hypothesis.

There was no significant difference between TA and SOL axons at any of the five depolarizing conditioning strengths. At hyperpolarizing conditioning strengths, however, we found that TA axons had significantly greater threshold increases at I/V -80% (*Z* = 3.07, *p* = 0.002) and -90% (*Z* = 2.98, *p* = 0.003; **Figure 3**).

The slopes of the TA and SOL I/V curves were calculated at hyperpolarizing conditioning strengths, and the slope of the TA axon line was found to be significantly smaller than the SOL axon line at I/V -40% (*Z* = 2.79, *p* = 0.045) and -50% (*Z* = 3.10, *p* = 0.018). The values for these I/V measurements are given in **Table 1**.

### RECOVERY CYCLE

**FIGURE 4:**
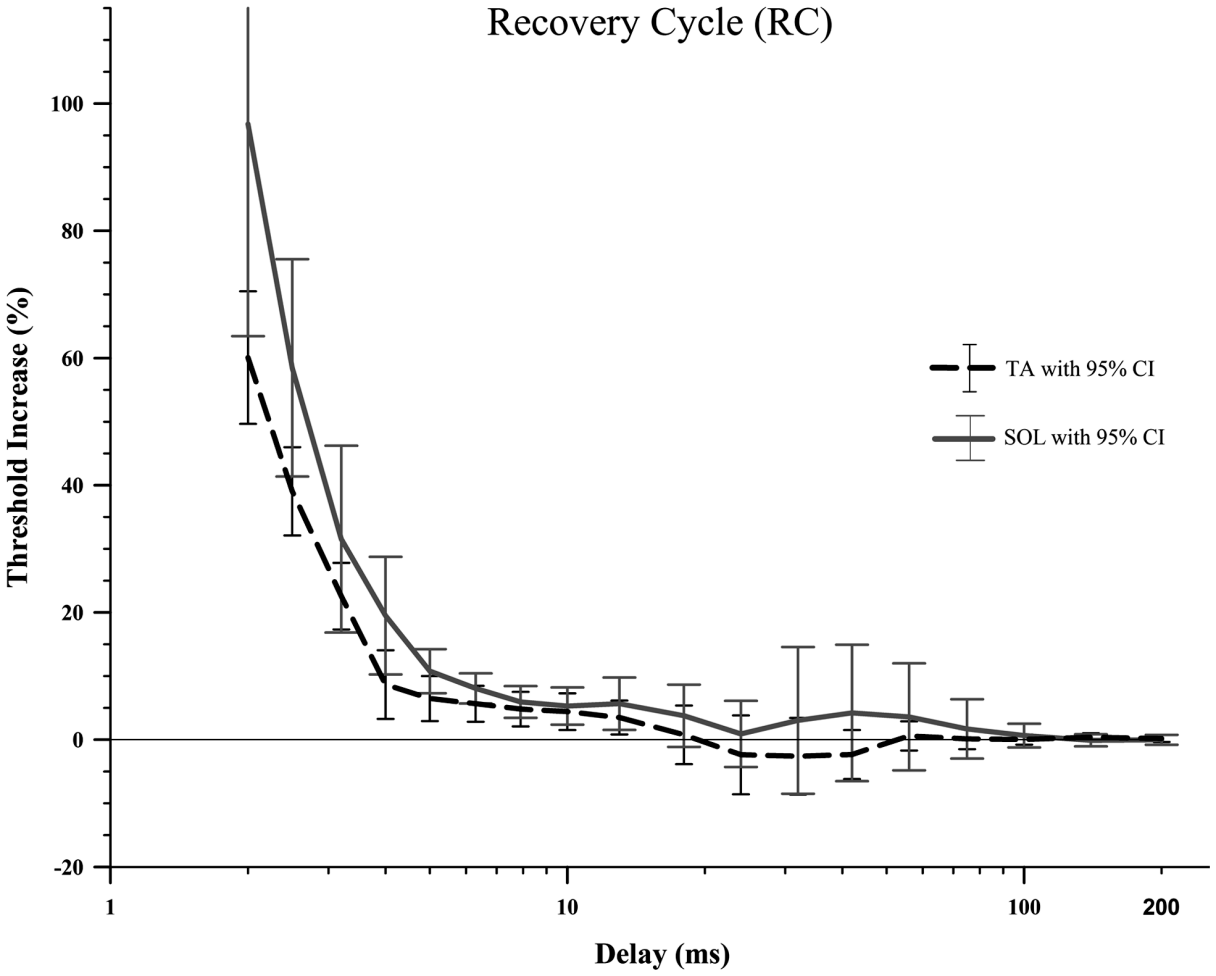
**The recovery cycle in the rat TA and SOL motor axons did not show a period of superexcitability followed by subexcitability, just a slowly decaying relative refractory period.** A Wilcoxon signed-rank test did not find any significant differences between TA and SOL axons at any of the 18 delays. It should be noted that the large number of delays (18) that were compared created a very small adjusted alpha level, 0.003. Therefore there is an increased risk of type 2 error.

There were no significant differences between TA and SOL axons in RC (**Figure 4**; **Table 1**) using the adjusted alpha value of 0.003 for multiple comparisons. Comparison at delays of 2–5 ms showed that TA axons had smaller increases in threshold compared to SOL axons (*p*-values less than 0.05), but these differences did not meet criteria for rejection of the null-hypothesis given the multiple comparisons.

### STIMULUS-RESPONSE CURVES, RHEOBASE, AND STRENGTH-DURATION TIME CONSTANT

**FIGURE 5:**
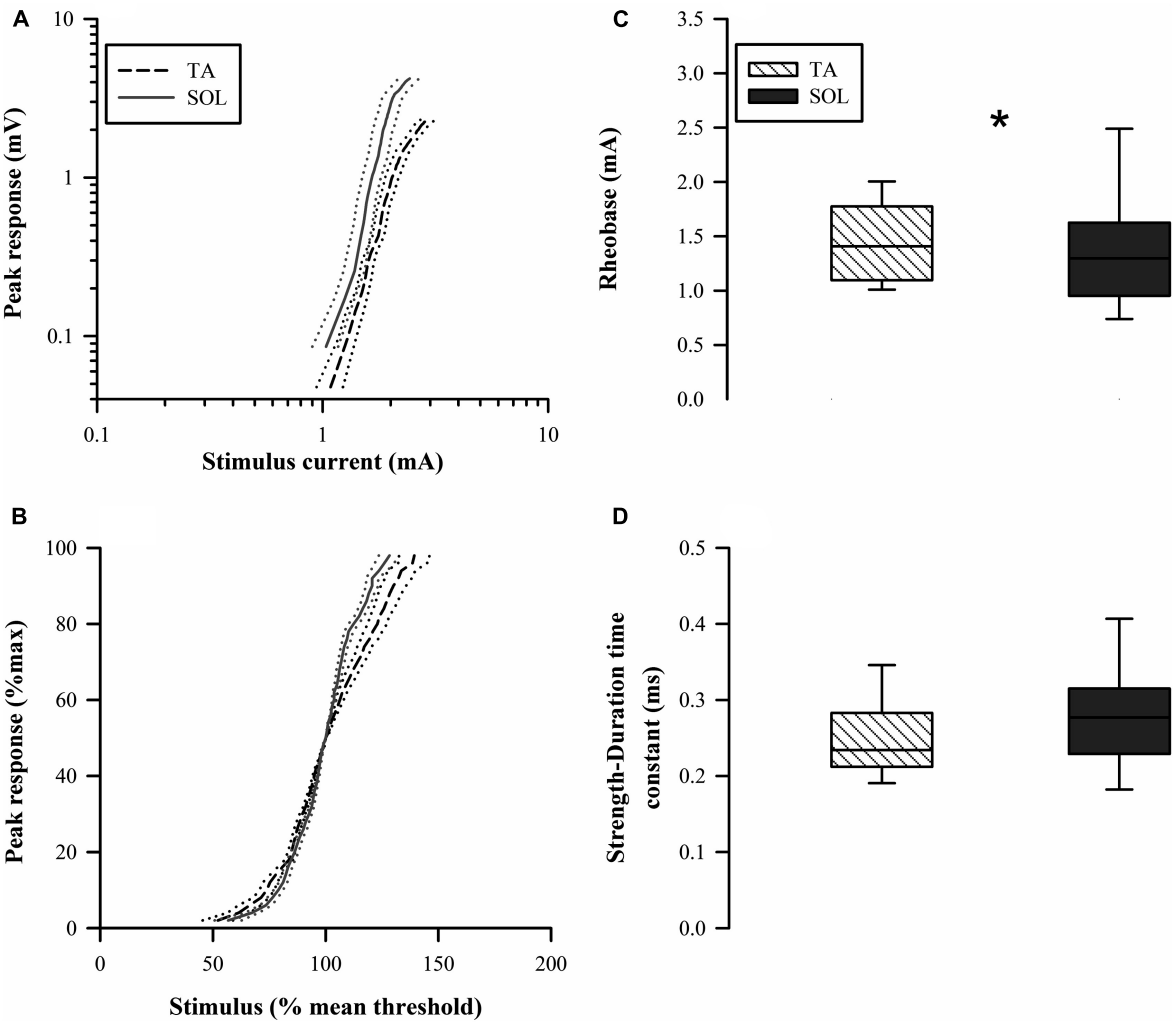
**Raw stimulus-response curves **(A)** are shifted to the right for TA axons indicating higher threshold.** The normalized stimulus-response curves **(B)** indicated that the relative slope is less for TA axons. **(C)** Rheobase for TA axons (median 1.45 mA) was significantly larger in paired comparisons to SOL axons (median 1.39 mA). **(D)** The strength-duration time constant (SDTC) was not significantly different for the two types of motor axons.

The stimulus-response curves showed a rightward shift for the TA axons indicating a higher threshold (**Figure 5A**). The normalized stimulus-response curve for TA was less steep compared to that for the SOL axons (**Figure 5B**). Paired comparisons found that TA axon rheobase was modestly but significantly larger than SOL axon rheobase (*Z* = 1.99, *p* = 0.047; **Figure 5C**). The higher rheobase for TA axons is consistent with the rightward shift of the stimulus-response curve. The SDTC was not different between the two axon groups (*Z* = 1.36, *p* = 0.17; **Figure 5D**).

## DISCUSSION

The present findings show that many measures of nerve excitability are indistinguishable for slow soleus and fast TA motor axons in the rat. The exception to this general finding is for measures of accommodation to prolonged hyperpolarization during the TE and I/V tests. The most parsimonious explanation for the differences is that voltage-gated inwardly rectifying current I_H_ is greater in slow compared to fast axons. However, some of the differences can not be explained by differences in I_H_: stimulus-response curves and rheobase, as well as differences in -40% TE at the early delay of 20–30 ms.

The most direct evidence implicating the channel responsible for I_H_, hyperpolarization-activated cyclic nucleotide-gated (HCN) channel, in TE and I/V nerve excitability measures comes from direct *in vitro* voltage recording of rat spinal root myelinated axons (Baker et al., 1987). HCN channels are blocked by cesium (Biel et al., 2009) and Baker et al. (1987) showed that the late accommodation in measures similar to TE and I/V was antagonized by cesium. Yang et al. (2000) used cesium chloride to decrease accommodation to hyperpolarizing TE measures using an *in vivo* rat, tail motor axon preparation similar to the current study. For TE -20 and -40% conditioning pulses the threshold reduction at delays of 100–109 ms was greater after cesium administration. Thus reducing I_H_ in mixed tail motor axons generates responses that are similar to the data from fast TA motor axons reported here (**Figure 2**). This supports our inference that fast motor axons have weaker I_H_ activity compared to slow motor axons.

This is not the first study to suggest that I_H_ may vary across a population of motor axons, but it is the first to test two clearly distinct populations. A previous study investigated motor axons in human median nerve to conclude that the lowest threshold axons had different electrophysiological properties compared to higher threshold axons (Trevillion et al., 2010). The best explanation for the unique properties of low-threshold motor axons was that they had a greater level of I_H_ activity, and this inference was based on computer modeling. The authors used *F*-wave latency to characterize ten single motor units (45% of their total sample) as having a faster CV and therefore larger diameter compared to the average motor axon in the population responses. Their conclusion was that axons of a larger diameter and faster CV had greater I_H_ activity. This is the opposite of the findings in the present study that found greater I_H_ activity in the slower soleus motor axons compared to fast TA motor axons. It is not clear at this time why there is a clear discrepancy in the two findings other than the obvious species difference and use of anesthetic.

There are four isoforms of the HCN channel, HCN1–HCN4, all of which allow for passage of a mixed inward cationic current of both K^+^ and Na^+^ ions. HCN channels are found in all three major neuron compartments: the soma, dendritic tree, and the axon proper (Baker et al., 1987; Takigawa et al., 1998; Robinson and Siegelbaum, 2003; Biel et al., 2009). In addition to being voltage dependent, HCN channels have been found to be influenced by acidic lipids (i.e., PIP2), protons, extracellular K^+^ concentration, and cytosolic proteins, and usually most significantly by cAMP (Biel et al., 2009). HCN1 has the fastest time constant of activation which can range anywhere from 30 to 200 ms at -140 to -95 mV. HCN2 is the next fastest isoform with the time constant of activation ranging from 150 ms to 1 s (Biel et al., 2009). Two recent nerve excitability studies comparing nerve excitability properties of motor and sensory axons have discussed the hypothesis that differences in I_H_ between these two axon groups are due to sensory axons having either greater expression of faster HCN isoforms and/or a different availability of HCN ligands that renders greater I_H_ in sensory compared to motor axons (Howells et al., 2012; Nodera and Rutkove, 2012). A higher density of channels on slow axons, the expression of different isoforms, or cAMP dependent phosphorylation status generating a difference in half-activation voltages could explain the increased I_H_ in slow motor axons in the present study. There was no *a priori* hypothesis that I_H_ current would dominate the difference between slow and fast axons. Future studies should use a modified nerve excitability testing protocol with additional stronger and more prolonged hyperpolarizing conditioning stimuli to further characterize the difference in I_H_ between fast and slow axons (Tomlinson et al., 2010).

Thus, it appears I_H_ is a plastic ionic conductance that varies according to the type of motor axon as well as between motor and sensory axons. As the type of activity patterns are different between most motor and sensory axons, as well as for lower and higher threshold axons, this points to the possibility that I_H_ expression depends on impulse activity patterns. This is the interpretation given for the finding of reduced I_H_ in human motor axons on the affected side for individuals experiencing hemiparesis subsequent to stroke (Jankelowitz et al., 2007). In these individuals the motor axons on the affected side are expected to have a chronic reduction in impulse activity, if spasticity is not excessive. The reduced chronic activity leads to an adaptive change in I_H_ that is consistent with our finding of less accommodation to hyperpolarization in the rat TA motor axons. Axons innervating the rat TA are mostly from fast motor units that fire less often but at higher frequencies than the axons of SOL, which are mostly from slow motor units (Gillespie et al., 1987; Totosy de Zepetnek et al., 1992; Gorassini et al., 2000). Activity-dependent hyperpolarization of axons is known to correlate positively with firing frequency as well as the number of impulses in a train (Erlanger and Gasser, 1973; Raymond, 1979; Morita et al., 1993; Kiernan et al., 2004), although the magnitude of hyperpolarization saturates at frequencies around 20–50 Hz in lizards (Morita et al., 1993) and possibly at 20–30 Hz in humans (Kiernan et al., 2004). Although the relative importance of firing frequency and impulse load in creating activity-dependent hyperpolarization in rat TA and SOL axons is unclear, (Raymond, 1979) has shown that in the frog, average firing frequencies as low as 1.25 Hz can cause axon threshold depression and therefore probably activity dependent hyperpolarization (see Figure 9 in Raymond, 1979). Bursts in rat SOL motor units fire at an average frequency of 20 Hz for roughly 30% of the day (Hennig and Lomo, 1985), and so it seems likely that SOL axons would generally experience a greater tendency toward activity-dependent hyperpolarization than TA axons due to greater activation by the Na^+^–K^+^ pump. It is possible that greater I_H_ expression in SOL axons offsets a tendency for their hyperpolarization caused by higher levels of activity.

While many of the differences in nerve excitability indices are consistent with the interpretation that I_H_ is greater in SOL axons, this mechanism may not explain differences in the stimulus-response curves, rheobase and early differences in hyperpolarizing TE. The finding that TA axons have a greater rheobase and have a rightward shift in the stimulus-response curve indicates that stronger stimuli were required to activate these axons. The magnitude of the differences was small and should be considered when assessing the importance of this finding. For example, rheobase differed on average by 0.06 mA that was much smaller than the SDs for this measure, which were 0.20 and 0.34 mA for TA and SOL axons respectively. Typically differences in rheobase have not been interpreted as resulting from I_H_. Similarly, the rate of activation of HCN channels is typically considered too slow to contribute to differences in hyperpolarizing TE at the 20–30 ms delay (Tomlinson et al., 2010). It is important to emphasize that the difference reported in **Figure 2B** was not a result of different extents of the fast change in threshold at the onset of the conditioning stimuli. The typical delay for overt expression of the effects of I_H_ is closer to 100 ms in human studies but can be much earlier if the membrane potential is depolarized (Bostock et al., 1998). There is some evidence from nerve excitability studies in mice that axons in the sciatic nerve may appear to be depolarized, even if there is no reason to suppose this to be the case (Boerio et al., 2009, 2010). In any case, it can reasonably be argued that if there is membrane depolarization, it should affect the TA and SOL axons equally and facilitate the measurement of greater I_H_ in SOL axons at earlier delays. Alternatively, differences in tissue impedance between rat and human experiments may facilitate earlier onset of I_H_ in rat experiments.

In conclusion, while for many nerve excitability measures we were not able to distinguish between slow and fast motor axons, they were significantly different during strong 100–200 ms hyperpolarizing conditioning stimuli. The axon threshold in these conditions is strongly affected by hyperpolarization-activated current I_H_ and the findings suggest a greater level of I_H_ in slow motor axons. This finding has implications for the use of nerve excitability testing in neurodegenerative conditions like amyotrophic lateral sclerosis (ALS). In the SOD1-G93A mouse model of ALS there is a clear motor unit type dependent vulnerability with fast-fatiguing motor units being most susceptible (Frey et al., 2000; Pun et al., 2006; Hegedus et al., 2008; Deforges et al., 2009; Kanning et al., 2010). As the vulnerable motor axons become denervated early in the disease the population of axons generating the nerve excitability measures will change. Longitudinal changes in nerve excitability measures (e.g., Kanai et al., 2006) may be confounded by the change in population of heterogeneous motor axons that contribute to the measure.

## AUTHOR CONTRIBUTIONS

Chad Lorenz contributed to the acquisition, analysis and interpretation of the data, drafted the initial version of the manuscript, derived from a Master’s thesis, and gave final approval for the submitted publication. Kelvin E. Jones conceived the study and contributed to acquisition, analysis, and interpretation of the data. The final manuscript was revised for publication by Kelvin E. Jones.

## Conflict of Interest Statement

The authors declare that the research was conducted in the absence of any commercial or financial relationships that could be construed as a potential conflict of interest.
